# Urea Synthesis and Excretion in *Aedes aegypti* Mosquitoes Are Regulated by a Unique Cross-Talk Mechanism

**DOI:** 10.1371/journal.pone.0065393

**Published:** 2013-06-05

**Authors:** Jun Isoe, Patricia Y. Scaraffia

**Affiliations:** Department of Chemistry and Biochemistry, The Center for Insect Science, The University of Arizona, Tucson, Arizona, United States of America; New Mexico State University, United States of America

## Abstract

*Aedes aegypti* mosquitoes do not have a typical functional urea cycle for ammonia disposal such as the one present in most terrestrial vertebrates. However, they can synthesize urea by two different pathways, argininolysis and uricolysis. We investigated how formation of urea by these two pathways is regulated in females of *A. aegypti*. The expression of arginase (AR) and urate oxidase (UO), either separately or simultaneously (ARUO) was silenced by RNAi. The amounts of several nitrogen compounds were quantified in excreta using mass spectrometry. Injection of mosquitoes with either dsRNA-AR or dsRNA-UO significantly decreased the expressions of AR or UO in the fat body (FB) and Malpighian tubules (MT). Surprisingly, the expression level of AR was increased when UO was silenced and vice versa, suggesting a cross-talk regulation between pathways. In agreement with these data, the amount of urea measured 48 h after blood feeding remained unchanged in those mosquitoes injected with dsRNA-AR or dsRNA-UO. However, allantoin significantly increased in the excreta of dsRNA-AR-injected females. The knockdown of ARUO mainly led to a decrease in urea and allantoin excretion, and an increase in arginine excretion. In addition, dsRNA-AR-injected mosquitoes treated with a specific nitric oxide synthase inhibitor showed an increase of UO expression in FB and MT and a significant increase in the excretion of nitrogen compounds. Interestingly, both a temporary delay in the digestion of a blood meal and a significant reduction in the expression of several genes involved in ammonia metabolism were observed in dsRNA-AR, UO or ARUO-injected females. These results reveal that urea synthesis and excretion in *A. aegypti* are tightly regulated by a unique cross-talk signaling mechanism. This process allows blood-fed mosquitoes to regulate the synthesis and/or excretion of nitrogen waste products, and avoid toxic effects that could result from a lethal concentration of ammonia in their tissues.

## Introduction

Mosquitoes constitute a severe scourge for the world population since they are vectors of etiological agents that cause more than one million deaths annually [1]–[5]. In recent years, the populations of *Aedes aegypti*, the main vector of dengue and yellow fever viruses, as well as the number of cases of people infected with viruses transmitted by these mosquitoes have increased dramatically [6], [7]. Therefore, the implementation of more effective strategies for mosquito control is necessary in order to reduce this devastating worldwide problem.

Blood feeding insects, including mosquitoes, must be able to deal with the potentially life-threatening overload of nitrogen that makes up a disproportionate amount of the nutrients in the blood meal. However, how the female mosquitoes can overcome this metabolic challenge during blood meal digestion is poorly understood. Metabolic labeling studies using ^14^C-proteins revealed that in *A. aegypti* most of the amino acids generated after the digestion of a blood meal (∼70%) are oxidized for immediate energy needs and excreted as CO_2_ or waste; 20% is retained in the female as a mixture of protein (∼10%), lipid (∼8%), and sugar (∼2%); whereas only 10% is allocated for oogenesis (∼4% for protein and ∼6% for lipid) [8].

We showed previously that *A. aegypti* females very efficiently detoxify ammonia, consisting of NH_3_ or NH_4_^+^ or a combination of the two, [9]–[13]. By applying direct infusion electrospray and tandem mass spectrometry methods, we reported that ammonia metabolism in *A. aegypti* whole body, tissues, and excreta occurs through three phases: fixation, assimilation and excretion [11]–[13]. It was also demonstrated that fat body and midgut use distinct metabolic pathways for metabolizing ammonia [12]. Thus, *A. aegypti* midgut mainly fixes and assimilates ammonia into glutamine and alanine by reactions catalyzed by glutamine synthetase (GS), glutamate dehydrogenase (GDH) and alanine aminotransferase (ALAT), whereas the fat body mainly fixes and assimilates ammonia into glutamine and proline by using a GS/glutamate synthase (GS/GltS) pathway, as well as pyrroline-5-carboxylate synthase (P5CS), pyrroline-5-carboxylate reductase (P5CR), GDH and ALAT [12]. Additionally, *A. aegypti* mosquitoes can use the amide group of glutamine to synthesize uric acid, and further excrete and metabolize it into allantoin, allantoic acid and urea through an amphibian-like uricolytic pathway, which involves three enzymes: urate oxidase, allantoinase and allantoicase [13]. Moreover, *A. aegypti* mosquitoes do not have a typical functional urea cycle for ammonia disposal such as the one present in most terrestrial vertebrates [14]. However, in addition to the utilization of uric acid for urea synthesis mentioned above, *A. aegypti* can also synthesize urea through a reaction catalyzed by arginase [15], [16], which uses arginine as a substrate either provided in the diet or from the turnover of endogenous proteins. The metabolic regulation of urea synthesis generated by argininolysis and uricolysis has not been investigated previously in any organism that has both functional pathways, and therefore this biological system has the potential to uncover new modes of metabolic regulation. We analyzed this process in *A. aegypti* mosquitoes by using multiple approaches, including reverse genetics and mass spectrometry, and demonstrated that urea synthesis and excretion in blood-fed *A. aegypti* females are tightly regulated by a unique cross-talk signaling mechanism.

## Results

### Arginase (AR) Expression is Modified when Urate Oxidase (UO) Expression is Silenced and Vice Versa

**Figure 1:**
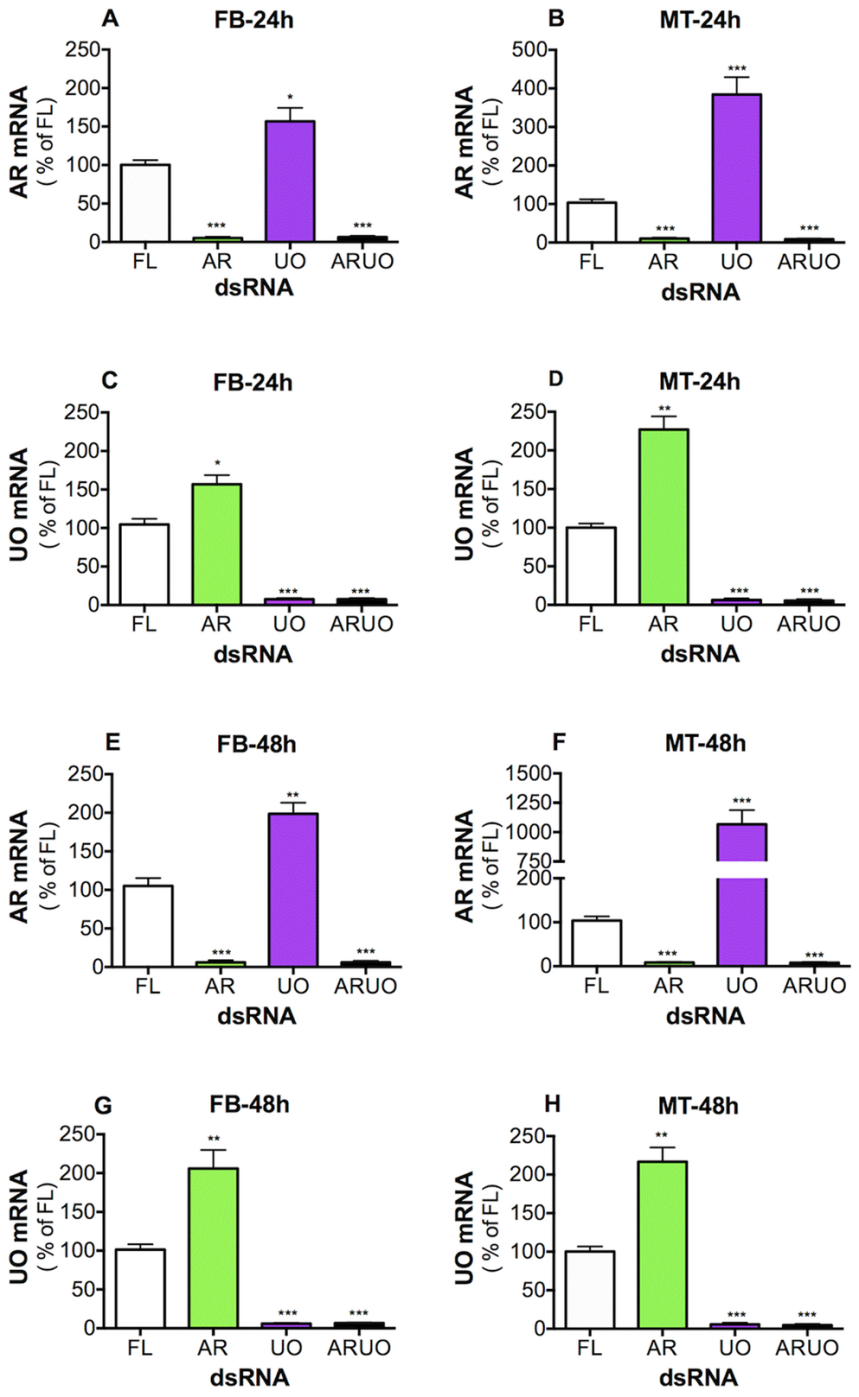
Effect of arginase (AR), urate oxidase (UO) and ARUO knockdown on gene expression. *A. aegypti* females were injected with dsRNA-firefly luciferase (dsRNA-FL), dsRNA-AR, dsRNA-UO or dsRNA-ARUO and then fed a blood meal. A-D. Relative abundance of AR and UO mRNA in tissues of dsRNA-injected females at 24 h after blood feeding. E–H. Relative abundance of AR and UO mRNA in tissues of dsRNA-injected females at 48 h after blood feeding. Data are presented as the mean ± SEM of five independent samples. *p<0.05, **p<0.01, ***p<0.001 (when compared to dsRNA-FL by ANOVA).

To assess the efficiency of dsRNA-mediated knockdown, arginase and urate oxidase expression was first evaluated by qRT-PCR in the fat body (FB) and Malpighian tubules (MT) of individual dsRNA-injected mosquitoes at 24 (Fig. 1 A–D) and 48 h after blood feeding (Fig. 1 E–H). We will refer to all genes, transcripts and enzymes for arginase and urate oxidase as AR and UO, respectively. *A. aegypti* females were first injected with dsRNA-firefly luciferase control (dsRNA-FL), dsRNA-arginase (dsRNA-AR), dsRNA-urate oxidase (dsRNA-UO) or both dsRNA-AR and dsRNA-UO (dsRNA-ARUO), and then fed a blood meal. As expected, AR expression was significantly reduced in FB and MT of both dsRNA-AR and dsRNA-ARUO-injected females at 24 h (Fig. 1 A–B) and 48 h after blood feeding (Fig. 1 E–F), when compared to dsRNA-FL-injected controls. UO expression also significantly decreased in both FB and MT of dsRNA-UO and dsRNA-ARUO-injected females at 24 h (Fig. 1 C–D) and 48 h after feeding (Fig. 1 G–H). These data indicate that dsRNA-mediated knockdown significantly silences the expression of AR and UO. However, one of the most surprising observations was that silencing of UO increased AR expression in both tissues (Fig. 1 A–B and E–F) and vice versa (Fig. 1 C–D and G–H). Knockdown of AR produced an almost two-fold increase of UO transcript in both tissues FB and MT at 24 or 48 h after feeding compared with mosquitoes injected with dsRNA-FL (Fig. 1 C–D and G–H). Furthermore, silencing of UO increased AR transcript levels at 48 h after feeding two-fold in FB (Fig. 1 E) to more than ten-fold in MT (Fig. 1 F) compared with control. The data suggest that a cross-talk between AR and UO occurs in mosquito tissues.

### AR and UO Regulate the Synthesis and Excretion of Urea and Other Nitrogen Compounds

**Figure 2:**
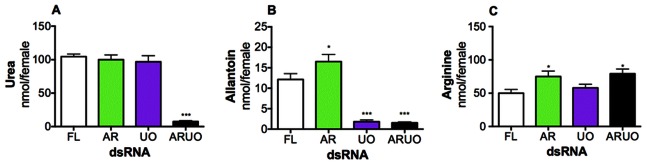
Effect of arginase (AR), urate oxidase (UO) and ARUO knockdown on nitrogen waste excretion. *A. aegypti* females were injected with dsRNA-firefly luciferase (dsRNA-FL), dsRNA-AR, dsRNA-UO or dsRNA-ARUO and then fed with a blood meal. A–C. Urea, allantoin and arginine concentrations measured in the female excreta by mass spectrometry at 48 h after blood feeding. Data are presented as the mean ± SEM of five independent samples. *p<0.05, ***p<0.001 (when compared to dsRNA-FL by ANOVA).

To functionally validate that synthesis and excretion of urea in mosquitoes are regulated by a cross-talk mechanism, nitrogen waste products were quantified in the excreta of mosquitoes that were injected with dsRNA and fed a blood meal. The effect of silencing AR, UO and ARUO on urea, allantoin and arginine excretion was studied (Fig. 2). At 48 h after blood feeding, the urea concentration in the excreta of females injected with dsRNA-AR or dsRNA-UO remained unchanged compared with control. However, the urea concentration decreased significantly in dsRNA-ARUO-injected mosquitoes (Fig. 2A). The allantoin concentration also decreased significantly in those mosquitoes injected with dsRNA-UO or dsRNA-ARUO, whereas a significant increase in allantoin concentration was observed in the excreta of dsRNA-AR-injected mosquitoes (Fig. 2B). In addition, arginine concentration in the excreta increased significantly in those females injected with dsRNA-AR or dsRNA-ARUO (Fig. 2C), indicating that arginine excretion contributes to the efficient elimination of excess nitrogen in blood-fed females. A similar pattern was also observed at 24 h after blood feeding (data not shown). These data demonstrate that both argininolysis and uricolysis pathways are finely interconnected and that AR and UO regulate the synthesis and excretion of urea and other nitrogen compounds in *A. aegypti* females.

### Arginase and Nitric Oxide Synthase Inhibition Affects the UO Expression and Nitrogen Waste Excretion

**Figure 3:**
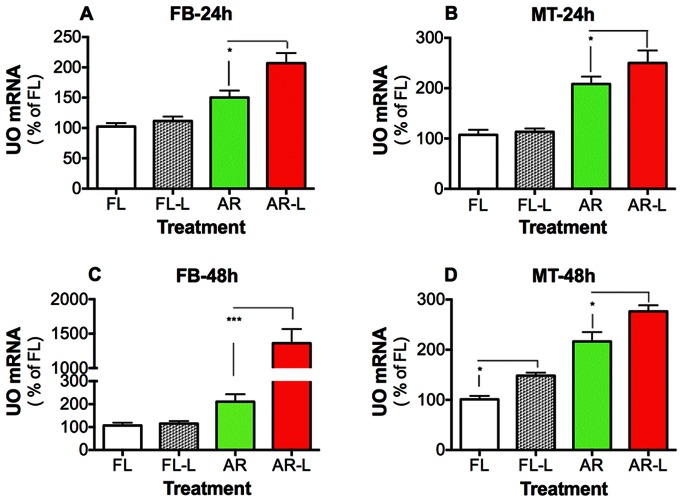
Effect of arginase (AR) knockdown and L-NAME, a nitric oxide synthase inhibitor, on urate oxidase (UO) expression. *A. aegypti* females were injected with dsRNA-firefly luciferase (dsRNA-FL) or dsRNA-AR and then fed with a blood meal in the presence or absence of L-NAME (L). A–B. Relative abundance of UO mRNA in tissues of injected mosquitoes at 24 h after blood feeding. C–D. Relative abundance of UO mRNA in tissues of injected mosquitoes at 48 h after blood feeding. Data are presented as the mean ± SEM of five independent samples. *p<0.05, ***p<0.001 (when compared to dsRNA-FL or dsRNA-AR by unpaired Student’s *t*-test).

**Figure 4:**
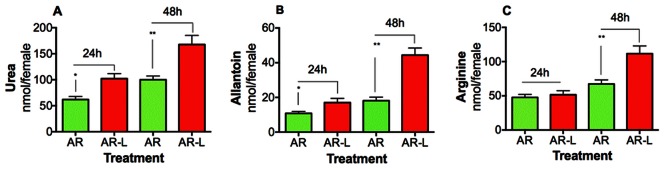
Effect of silencing arginase (AR) and nitric oxide synthase on nitrogen waste excretion. A–C. Urea, allantoin, and arginine concentrations quantified by mass spectrometry at 24 and 48 h after feeding dsRNA-AR-injected mosquitoes with a blood meal and L-NAME (L), a nitric oxide synthase inhibitor. Data are presented as the mean ± SEM of five independent samples. *p<0.05, **p<0.01 (when compared to dsRNA-AR by unpaired Student’s *t*-test).

To provide further evidence that urea synthesis and excretion in mosquitoes are cross-regulated, we next explored whether uricolysis and nitrogen waste excretion can be modified by simultaneously inhibiting argininolysis and nitric oxide synthase (NOS). The effect of knocking down AR in the presence of a NOS inhibitor was studied (Fig. 3). In mosquitoes injected with dsRNA-FL (control) and fed with blood and N_ω_-Nitro-L-arginine methyl ester (L-NAME), the expression of UO was only modified in the MT at 48 h after feeding (Fig. 3D). Interestingly, at 24 h after feeding the expression of UO increased significantly in FB and MT of those blood-fed mosquitoes in which both AR and NOS were inhibited (Fig. 3 A–B). The changes of UO expression in FB were also evident at 48 h after feeding, where the expression of UO increased about 10-fold in females injected with dsRNA-AR and fed with NOS inhibitor (Fig. 3 C–D). These results confirm that AR silencing and NOS inhibition strongly affect UO transcript. Furthermore, to verify that uricolytic pathway is functionally stimulated when AR and NOS are inhibited, we monitored the concentrations of nitrogen waste products in the mosquito excreta (Fig. 4). The urea and allantoin amount in the excreta of females injected with dsRNA-AR and fed with blood meal containing L-NAME, increased significantly at 24 h and 48 h after feeding, compared with control (Fig. 4 A–B). At 48 h after feeding, the allantoin concentration in the excreta of those mosquitoes in which both AR and NOS were inhibited increased almost three-fold. These findings indicate that the simultaneous inhibition of AR and NOS activates the uric acid catabolism. In addition, it is noticeable that at 48 h after feeding, arginine concentration rose in the excreta of those mosquitoes injected with dsRNA-AR and fed with blood supplemented with NOS inhibitor (Fig. 4C). This process could facilitate the elimination of an excess of arginine that cannot be utilized for protein synthesis or metabolized into other compounds. These data support the results described above and taken together suggest that in addition to arginase and urate oxidase, nitric oxide synthase also plays a role in the metabolic regulation of urea in blood-fed female mosquitoes.

### The Silencing of AR and UO Causes a Transient Delay of Digestion and Vitellogenesis

**Figure 5:**
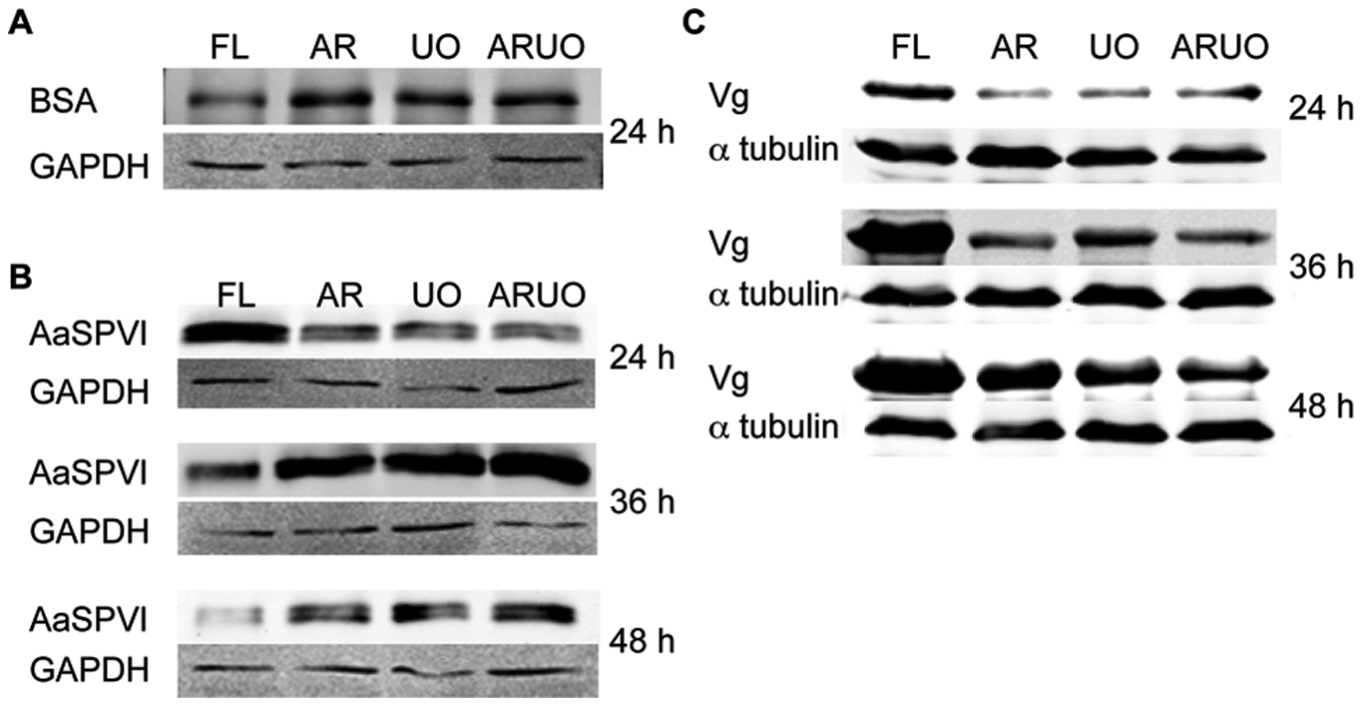
Effect of arginase (AR) and urate oxidase (UO) deficiency on blood meal digestion and ovarian development. *A. aegypti* females were injected with dsRNA targeting AR, UO or both (ARUO). *A. aegypti* females injected with dsRNA-firefly luciferase (dsRNA-FL) were used as a control. A. Representative western blot analysis of the level of intact BSA remaining 24 h after blood feeding in the midgut of mosquitoes injected with dsRNA. Proteins were extracted from midgut. Each lane was loaded with 0.2 midgut equivalent of the protein extracts. Protein loading was monitored using an anti-GAPDH antibody. B. Representative western blot analysis of the level of AaSPVI trypsin 24, 36, and 48 h after blood feeding in the midgut of mosquitoes injected with the dsRNA. Proteins were extracted from the midgut. Each lane contains 0.2 midgut equivalent of the protein extracts. Western blotting was performed using the antigen-specific AaSPVI antibody. An anti-GAPDH antibody was used to control protein loading. C. Representative western blot analysis of the level of vitellogenin in the ovaries of mosquitoes injected with dsRNA. Mosquitoes were dissected at 24, 36, and 48 h after blood feeding. Proteins were extracted from the ovaries. Each lane contains 0.3 ovary equivalent of the protein extracts. Western blotting was performed using a monoclonal antibody against *A. aegypti* vitellogenin. Protein loading was monitored using an anti-α-tubulin antibody.

To evaluate whether the massive reduction of argininolysis and/or uricolysis through RNA interference affects the digestion of the blood, the pattern of blood digestion in the midgut was examined through monitoring the level of two reference proteins: bovine serum albumin (BSA), an abundant blood protein, and AaSPVI, a major trypsin secreted from mosquito midgut epithelial cells into the lumen during blood protein digestion [17]–[18]. Total RNA was isolated from FB and MT from individual blood-fed females injected with dsRNA-FL, AR, UO or ARUO, and subjected to qRT-PCR to evaluate the knockdown efficiency. The proteins extracted from the midgut and ovaries from the same mosquito were then used for western blot analyses with antibodies against BSA, AaSPVI and vitellogenin. Interestingly, at 24 h after blood feeding the amount of intact BSA remaining in the midguts from mosquitoes injected with either dsRNA-AR, dsRNA-UO or dsRNA-ARUO-injected blood-fed females was higher than that of dsRNA-FL-injected controls, suggesting that the reduced level of either arginase or urate oxidase or both delayed the blood protein digestion in the midgut (Fig. 5A). In correlation with these data, at 24 h after feeding, a low amount of AaSPVI trypsin was observed in the midguts of those females injected with dsRNA against arginase and urate oxidase genes (Fig. 5B). By 36 h, the protein level of AaSPVI had increased significantly, and it remained abundant by 48 h after feeding compared to the dsRNA-FL- injected control mosquitoes, in which the digestion process is almost completed.

Since the blood meal digestion was delayed in mosquitoes injected with dsRNA-AR, dsRNA-UO or both (dsRNA-ARUO), we next examined the effect of knockdown on ovarian development by measuring the vitellogenin protein levels in the ovaries (Fig. 5C). The uptake of the vitellogenin by the ovaries of the AR, UO or ARUO dsRNA-injected females occurred at a lower rate during the first 48 h compared to the control. Mosquitoes injected with dsRNA against arginase and urate oxidase completed digestion and matured their oocytes by 72 h after feeding. Taken together, these data clearly show that knockdown of genes involved in argininolysis and uricolysis leads to a temporary delay in both physiological processes: digestion and vitellogenesis.

### The Silencing of Argininolysis and Uricolysis Affects the Expression of Genes Involved in Ammonia Metabolism

**Figure 6:**
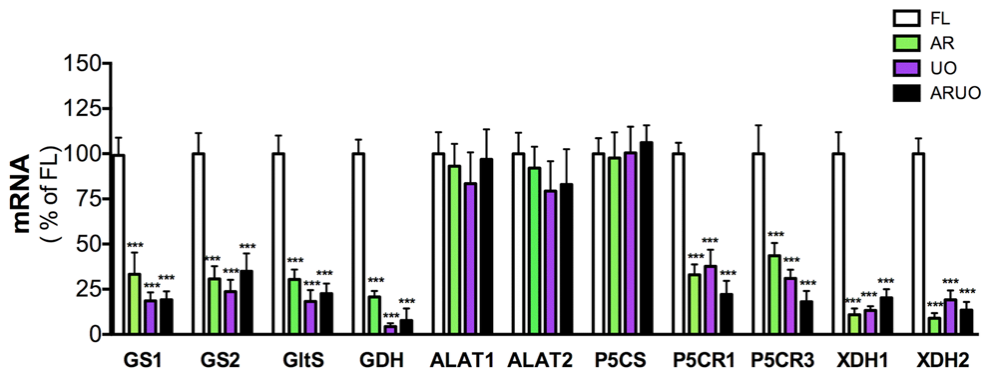
Effect of silencing of arginase (AR), urate oxidase (UO) or both (ARUO) on the expression of several genes. Relative abundance of glutamine synthetase (GS1 and GS2), glutamate synthase (GltS), glutamate dehydrogenase (GDH), alanine aminotransferase (ALAT1 and ALAT2), pyrroline-5-carboxylate synthase (P5CS), pyrroline-5-carboxylate reductase (P5CR1 and P5CR3), xanthine dehydrogenase (XDH1 and XDH2) mRNA levels in the fat body of dsRNA-injected females at 48 h after feeding a blood meal. Data are presented as the mean ± SEM of five to ten independent samples. ***p<0.001 (when compared to dsRNA-firefly luciferase (dsRNA-FL) by ANOVA).

The transient delay in digestion and vitellogenesis described above led us to hypothesize that the synthesis and/or excretion of nitrogen waste regulate the expression of several genes involved in fixation, assimilation and excretion of ammonia in *A. aegypti* females. To verify this hypothesis, the expression patterns of genes encoding glutamine synthetase, (GS1 and GS2), glutamate synthase (GltS), glutamate dehydrogenase, (GDH), alanine aminotransferase (ALAT1 and ALAT2), pyrroline-5-carboxylate synthase (P5CS), pyrroline-5-carboxylate reductase (P5CR1, P5CR2 and P5CR3) and xanthine dehydrogenase (XDH1 and XDH2) were investigated. The levels of expression of these genes were analyzed in FB from AR, UO or ARUO dsRNA-injected females at 48 h after blood feeding. Our results demonstrate that the silencing of AR, UO and ARUO expression led to a large decrease in the mRNA levels for GS1, GS2, GltS, GDH, P5CR1, P5CR3, XDH1 and XDH2 at 48 h after feeding (Fig. 6). P5CR2 exhibited a similar expression pattern as P5CR1 (data not shown). No significant differences were observed in the expression of ALAT1, ALAT2 and P5CS when AR, UO or ARUO were silenced (Fig. 6). These data confirm that synthesis and/or excretion of nitrogen waste in mosquitoes are finely regulated by a delicate and complex cross-talk mechanism. By using this molecular mechanism, blood-fed female mosquitoes can control the regulation of nitrogen waste at the synthesis and/or excretion levels without affecting their survival.

## Discussion

Blood-fed *A*. *aegypti* females are able to fix, assimilate and excrete nitrogen very efficiently by using multiple metabolic pathways [9]–[13]. During this metabolic challenge, female mosquitoes excrete nitrogen waste products such as ammonia, uric acid, allantoin, allantoic acid and urea. Two metabolic origins have been proposed for the urea production, either from argininolysis [15], [16] or from uricolysis [13]. The argininolysis is catalyzed by arginase, which hydrolyzes arginine into ornithine and urea. The uricolysis refers to the hydrolysis of uric acid into glyoxylic acid and two urea molecules and takes place by reactions catalyzed successively by urate oxidase, allantoinase and allantoicase. A single-copy of each gene encoding enzymes involved in uric acid catabolism was previously identified and studied in *A. aegypti* tissues [13]. In this report, we show molecular and biochemical evidence that both pathways regulate the synthesis and excretion of urea by a cross-talk signaling mechanism.

As indicated in the Results section, mRNA levels of arginase (AR) and urate oxidase (UO) in *A. aegypti* fat body and Malpighian tubules at 24 and 48 h after blood feeding decreased significantly after silencing each of those genes in individual mosquitoes through RNAi. However, an unexpectedly high level of AR transcript was observed when UO was silenced and vice versa. Although the total amount of excreted urea decreased significantly in dsRNA-ARUO-injected mosquitoes, the effect of silencing individually each gene surprisingly did not result in a significant decrease in the total amount of urea excreted at 48 h after feeding, indicating that urea was still produced by one of the two pathways most likely regulated via cross-talk between argininolysis and uricolysis. In addition, allantoin concentration increased significantly in those mosquitoes injected with dsRNA-AR, indicating that the flux through the uricolytic pathway depends on the function of both arginase and urate oxidase. The decrease in allantoin concentration observed in those dsRNA-ARUO or dsRNA-UO-injected females is consistent with previously reported data that demonstrate when UO is silenced, the levels of uric acid temporarily increase in whole bodies of both blood-fed or ^15^NH_4_Cl-fed mosquitoes at 24 and 48 h after feeding, whereas the levels of ^15^N-urea decrease significantly [13]. In data reported here, we also provide biochemical evidence that the concentration of arginine excreted was not modified in dsRNA-UO-injected mosquitoes. However, arginine increased significantly when AR or ARUO were knocked down, most likely to facilitate the elimination of excess nitrogen.

**Figure 7:**
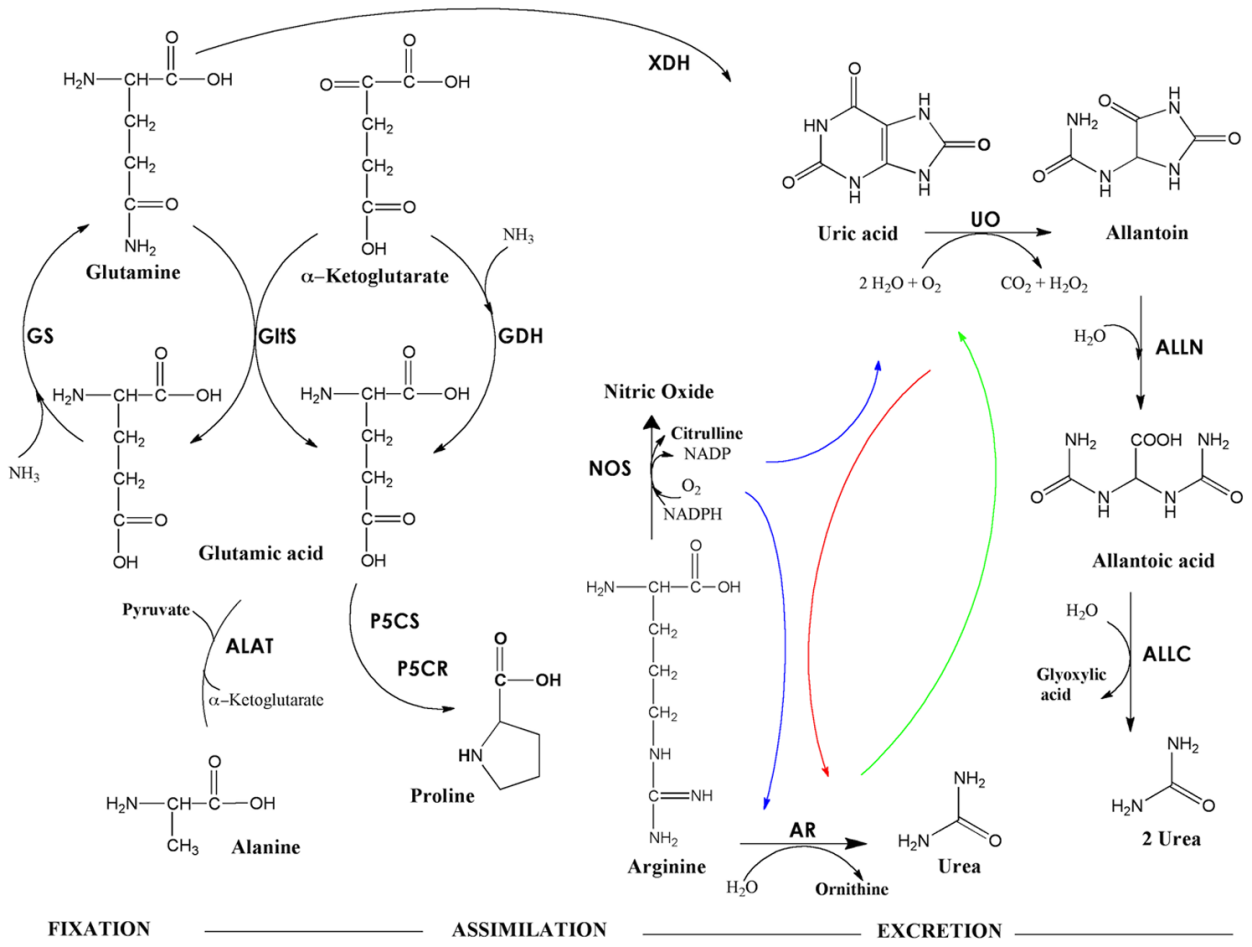
Integrated schematic representation of ammonia metabolism and proposed cross-talk mechanism for metabolic regulation of urea. Fixation, assimilation and excretion pathways of ammonia metabolism were previously studied in *A. aegypti* (11–13). The proposed metabolic regulation of urea occurs mainly via a cross-talk signaling mechanism. It affects the metabolism of other nitrogen compounds, as discussed in the text. Colored arrows indicate a cross-talk between UO (red), AR (green) and NOS (blue). Abbreviations: Glutamine synthetase (GS), glutamate synthase (GltS), glutamate dehydrogenase (GDH), alanine aminotransferase (ALAT), pyrrolidine-5-carboxylate synthase (P5CS), pyrrolidine-5-carboxylate reductase (P5CR), xanthine dehydrogenase (XDH), urate oxidase (UO), allantoinase (ALLN), allantoicase (ALLC), arginase (AR) and nitric oxide synthase (NOS).

A cross-talk signaling mechanism is proposed for the metabolic regulation of urea synthesis and excretion in *A. aegypti* mosquitoes (Fig 7). The existence of the proposed mechanism is supported by the results discussed above and the data obtained when dsRNA-injected mosquitoes were fed in the presence of L-NAME, a competitive inhibitor of nitric oxide synthase [19], [20]. Indeed, at 24 and 48 h after feeding, the level of UO mRNA was notably high in fat body and Malpighian tubules from dsRNA-injected mosquitoes that were fed with blood meal in the presence of nitric oxide synthase (NOS) inhibitor. In agreement with these data, the concentrations of urea, allantoin and arginine in the excreta from those mosquitoes increased significantly. We previously observed that the UO gene is induced not only by blood meal but also by ammonia [13]. The increased expression of UO mRNA in the presence of NOS inhibitor together with the increase of allantoin and urea in the excreta observed here demonstrate an active flux through the uricolytic pathway under those experimental conditions. Furthermore, the increase of arginine excretion confirms the presence of a protection mechanism of removing the excess nitrogen. Taken together, the data clearly demonstrate that a tight metabolic regulation for nitrogen disposal exists in *A. aegypti* females and that NOS is also involved in this process.

It is also noteworthy that the RNAi-mediated silencing of AR or UO individually or together causes modest phenotypical effects in mosquito females. In correlation with this observation, *Drosophila melanogaster* carrying a mutation of the arginase gene do not display a severe phenotype [21]. Although a developmental delay is associated with the mutation, the mutation does not affect viability. The flies survive normally, suggesting that the arginase gene does not provide a vital function. The functional role of the arginase in *Drosophila* may be the marginal benefit type but critical enough that it has been maintained during evolution [21]. On the other hand, a functional uricolytic pathway has been lost in the *Drosophila* lineage due to mutations in allantoinase and allantoicase [22], suggesting that mosquitoes may have evolved and adapted to efficiently utilize both argininolysis and uricolysis for elimination of nitrogen waste during acquisition of their blood feeding habits.

According to our present data, the transiently-delayed digestion of the blood and the delay in the uptake of vitellogenin in response to the silencing of AR, UO or ARUO expression in *A. aegypti* have a notable biological importance because they are implicated in the metabolic regulation of urea and other nitrogen waste products (Fig. 7). This assumption is supported by a significant decrease in the mRNA levels of GS, GltS, GDH, P5CR and XDH observed in fat body from AR, UO and ARUO dsRNA-injected females. As previously reported, fat body and midgut from *A. aegypti* fix ammonia released from the digestion of the blood meal mainly by GS [12]. GS produces glutamine that can be assimilated by GltS in fat body to generate glutamic acid, which in turn can be utilized to synthesize several other amino acids, such as alanine and proline (Fig. 7). GDH also plays a role in the glutamic acid synthesis [10]–[12]. Moreover, glutamine can act as a precursor for uric acid [13]. It is well known that the last two steps of uric acid synthesis in mosquitoes are catalyzed by XDH [15], [16]. Furthermore, uric acid can be excreted directly or degraded by uricolysis to produce allantoin, allantoic acid and then urea [13]. Via argininolysis, arginase cleaves arginine into urea and ornithine, which can be further utilized to synthesize several amino acids including proline. Thus, a temporarily hindered blood meal digestion in the midgut caused by the silencing of AR, UO or both (ARUO) in *A. aegypti* females leads to a delay in the release of ammonia from the ingested blood, as well as in the fixation, assimilation and excretion phases that take place during ammonia metabolism (Fig. 7). This process allows blood-fed female mosquitoes to regulate the synthesis and/or excretion of nitrogen waste, and avoid toxic effects that could result from a lethal concentration of ammonia in their tissues.

Collectively, the results presented in this study demonstrate that in *A. aegypti* mosquitoes the synthesis of urea by argininolysis and uricolysis is tightly-regulated and interconnected at the molecular level by a cross-talk signaling mechanism. This sophisticated metabolic regulation of urea directly affects the fixation, assimilation and excretion phases of ammonia metabolism, as well as the synthesis and/or excretion of other nitrogen waste products. These novel findings contribute to a better understanding of the molecular and biochemical mechanisms responsible for the success of the blood-fed female mosquitoes in their disposal of excess nitrogen and therefore their survival.

## Materials and Methods

### Rearing Mosquitoes

*A. aegypti* (NIH-Rockefeller strain) colony was maintained at 28°C, 75% relative humidity with a light: dark cycle of 16 h: 8 h. After the eggs were hatched, the larvae were maintained on a diet consisting of FormulaLab 5008 (Newco Distributors, Inc., Rancho Cucamonga, CA), fish food (goldfish flakes and tropical flakes in a proportion of 1∶3; Tetra, Blacksburg, VA) and liver powder (MP Biomedicals, Cleveland, OH) in a proportion of 10∶10:1. Male and female pupae were separated on the basis of their size using a separator model 5412 produced by John Hock Company (Gainesville, FL). Adult mosquitoes were kept in a CARON 6015 Insect Growth Chamber connected to a CARON CRSY 102 condensate recirculating system (Caron Products & Services, Inc., Marietta, OH) at 28°C, 75% relative humidity, and on a photoperiod of 16∶8 (L: D) h until the end of the experiments.

### Sample Preparation for Mass Spectrometry Analysis

Mated females were allowed to feed on a 3% sucrose solution or a bovine blood meal (Pel-Freez Biologicas, Rogers, AR), supplemented with ATP (5 mM) for 15 minutes. In some experiments, mosquitoes were injected with dsRNA and allowed to feed on a 3% sucrose solution supplemented with 1 mg/ml of N_ω_-Nitro-L-arginine methyl ester (L-NAME), a NOS inhibitor (Sigma-Aldrich, St. Louis, MO), and then they were fed a blood meal supplemented with ATP (5 mM) and L-NAME (1 mg/ml of blood). An inert stereoisomer, D-NAME (Sigma-Aldrich), was used as a negative control [19]. The mosquito excreta were prepared for mass spectrometry analysis as previously described [13], [23].

### Electrospray Ionization Tandem Mass Spectrometry

The mosquito excreta were electrosprayed into an AB/SCIEX 4000 QTRAP mass spectrometer (Applied Biosystems, Foster City, CA) and the quantification of nitrogen waste products was performed by electrospray ionization multiple reaction monitoring as previously reported [13], [23].

### Microinjection of Double-stranded RNA and qRT-PCR Assays

A gene encoding urate oxidase (UO, VectorBase AAEL002194) was previously studied in *A. aegypti* [13]. In this report, a putative ortholog of the mosquito arginase gene (AR, VectorBase AAEL002675) was identified by BLAST searches using fruit fly arginase (FBpp0070083-PA as a query [21]). The arginase gene in *A. aegypti* is a single copy gene. It encodes a protein of 349 residues and shares 85%, 43% and 54% identity to *Anopheles gambiae*, *D. melanogaster*, and *Bombyx mori*, respectively.

UO, AR and firefly luciferase (FL, GenBank accession number U47295) gene-specific primers flanked with T7 promoter sequence (Table 1) were used to PCR amplify DNA from mosquito cDNA and pGL3 vector (Promega, Madison, WI). Double-stranded RNA (dsRNA) covers approximately the first half of the coding sequence of UO or AR genes, and the target region corresponds to the catalytic domain of each protein. The dsRNA was prepared as described previously [17]. Newly-eclosed females were injected with 500 ng of dsRNA using a Nanoject II microinjector (Drummond Scientific Company, Broomall, PA) [13]. Females were fed with a blood meal 4 days later.

**Table 1:** Primers for qRT-PCR and dsRNA synthesis.

Genes		Primer sequence (5' to 3')	PCR, bp
*Gene-specific primers used for qRT-PCR*			
Glutamine synthetase 1 (AF004351)	Forward	CCTCTATGCTGGAGTTGACT	
	Reverse	CGCATCTGCTTGGTGGAGA	237
Glutamine synthetase 2 (AY623406)	Forward	GAGGAGTTTGGCATCGTTG	
	Reverse	GGTCGTAGGCTCGGATATG	181
Glutamate synthase (DQ383822)	Forward	CTCCTACAATACGGCATTCCAAC	
	Reverse	CGTGATCCGAGTAACTTCTTCTG	276
Glutamate dehydrogenase (AY623405)	Forward	GGCGAGAACCTGATGTACGA	
	Reverse	GAGCAGGTGGTAGTTGGACT	300
Alanine aminotransferase 1 (XM_001660422)	Forward	CAACTGCCGGAGAAGGCAAT	
	Reverse	TGATAGGTTCCATCCTTCTGACC	140
Alanine aminotransferase 2 (XM_001660420)	Forward	TTCTATGCATTCCAGCTGTTAGAGCA	
	Reverse	ACGCCCGGAACATGTCGAG	148
Pyrroline-5-carboxylate synthase (AY623404)	Forward	GCGGGAAGGTGTTAAGATCA	
	Reverse	GCAGCCGAATCATTCTCAGT	207
Pyrroline-5-carboxylate reductase 1 (AY623401)	Forward	GTGAACGAAGCACAGATGGAT	
	Reverse	TGCAGCTCCCATAACTGTTTG	159
Pyrroline-5-carboxylate reductase 2 (AY623402)	Forward	GCACGCGTCATCAGGGTCA	
	Reverse	CCTCAATCATGGTGAACACATA	217
Pyrroline-5-carboxylate reductase 3 (AY623403)	Forward	GCCGATGGAGGCGTAAGAAT	
	Reverse	GGCTGGGCTCGTTACATCATC	141
Xanthine dehydrogenase 1 (XM_001662081)	Forward	GCGATTGACATTGGACAGATCGA	
	Reverse	CCAGGAATGTCGGCGAAACC	146
Xanthine dehydrogenase 2 (XM_001648813)	Forward	GTTATGGACATTGGCTCTAGCCT	
	Reverse	CCTGGACCTCTCGATAGCAGTGT	140
Urate oxidase (EF676030)	Forward	CAGTCGGCGTTCGTGAACTT	
	Reverse	CCAGCAGAAATCGAAATCCAC	135
Arginase (XM_001662007)	Forward	AAGGAATTGGCGGATTACTGGC	
	Reverse	GCGTGGATACCGAACTTCTCAAT	143
Ribosomal protein S7 (AY380336)	Forward	ACCGCCGTCTACGATGCCA	
	Reverse	ATGGTGGTCTGCTGGTTCTT	131
*Gene-specific primers used for RNAi*			
Urate oxidase	Forward	*ATGATGTCCAGAAAGCTGGTTGA	
dsRNA	Reverse	*GAACGGTACTCATCGTTGAC	578
Arginase	Forward	*TGCTTCAGAACGGTAGTCGCCA	
dsRNA	Reverse	*ATCGGCTCCTGCCAATCCAT	562
Luciferase, pGL3-Basic Vector	Forward	*AGCACTCTGATTGACAAATACGA	
dsRNA	Reverse	*AGTTCACCGGCGTCATCGTC	548

* T7 bacteriophage promoter sequence (5' TAATACGACTCACTATAGGGAGA 3') was added in 5' of each primer.

The FB and MT were dissected from individual mosquitoes at 24 h and 48 h after blood feeding. Total RNA was extracted using TRIzol reagent (Life Technologies, Carlsbad, CA) and reverse transcribed using oligo-(dT)_20_ primer and reverse transcriptase (Promega, Madison, WI). The cDNA was then used as a template for qRT-PCR assays using gene-specific primers (Table 1). UO and AR knockdown efficiency, as well as the relative mRNA level of several other genes involved in nitrogen metabolism in *A. aegypti* were analyzed by qRT-PCR. Briefly, qRT-PCR was performed with PerfeCTa SYBR Green FastMix, ROX (Quanta BioSciences, Gaithersburg, MD) and a final primer concentration of 200 nM using Applied Biosystems 7300 Real-Time PCR System (Life Technologies, Carlsbad, CA) and the following PCR conditions: 95°C for 2 minutes followed by 40 cycles of 95°C for 10 seconds and 60°C for 30 seconds. The PCR efficiency of a primer set for each gene were verified by performing a dilution series experiment with a corresponding cloned plasmid DNA. Relative level of expression for each gene was calculated using the ΔΔC_T_ quantification method [24]. Ribosomal protein S7 transcript levels were used as an internal control for normalization of mRNA yields in all samples. The knockdown efficiency was determined as previously described [13].

### Protein Extraction and Western Blotting Procedures

For each mosquito, protein extracts from the dissected midgut and ovary were used for western blot analysis, while total RNA isolated from FB was used to determine the knockdown efficiency for RNAi as described above. Individual midguts and ovaries from dsRNA-injected females were dissected in 1X PBS at 24, 36, and 48 h after blood feeding. Tissues were homogenized using disposable hand homogenizers in a protein lysis buffer (1X PBS, 1% Triton X-100, 12 mM sodium deoxycholate, and 2% SDS) containing Complete, Mini, EDTA-free Protease Inhibitor Cocktail (Roche Applied Science, Mannheim, Germany). The protein extracts were heat-denatured and microcentrifuged at 14,000×g for 5 min, and the supernatant was stored at −80°C until used. For the single mosquito western blot analysis, the protein extracts from midgut and ovaries were subjected to SDS-PAGE only after confirmation that the corresponding FB from the same mosquito had over 85% knockdown. Western blot analysis was performed on 12% SDS-PAGE gels. The resolved proteins were electrophoretically transferred to a nitrocellulose membrane (Odyssey Nitrocellulose, LI-COR Biosciences, Lincoln, NE). The membranes were incubated with Qentix Western Blot Signal Enhancer (Thermo Scientific, Waltham, MA) followed by primary antibodies [17]. NewBlot Nitrocellulose Stripping Buffer (LI-COR Biosciences, Lincoln, NE) was used to remove the immunoreactive signals from the membranes and re-incubated with other primary antibodies used for loading controls. The dilutions of the primary antibodies were as follows: AaSPVI (1∶1000), BSA (1∶1000), GAPDH (1∶300), vitellogenin (1∶5000) and α-tubulin (1∶1000). Secondary antibodies used were either IRDye 800CW goat anti-rabbit (1∶10,000, LI-COR Biosciences, Lincoln, NE), IRDye 800CW goat anti-mouse, or IRDye 680LT monkey anti-chicken (1∶10,000, LI-COR Biosciences, Lincoln, NE) for 1 h. The immunoreactive protein bands were visualized with an Odyssey infrared imaging system (LI-COR Biosciences, Lincoln, NE). Anti-BSA polyclonal antibody was purchased from Gallus Immunotech (Cary, NC). Anti-GAPDH antibody was obtained from Cell Signaling Technology, Inc. (Danvers, MA). Anti-α-tubulin was obtained from Developmental Studies Hydridoma bank (University of Iowa, IA).

### Statistical Analysis

Unpaired Student’s *t*-test and one way analysis of variance (ANOVA) followed by Dunnett's Multiple Comparison Test were used. A *p*-value less than 0.05 was considered significant. All the statistical analyses were carried out using GraphPad Prism version 5.00 for Mac OS X (GraphPad Software, San Diego, CA).
